# The Lectin Pathway of Complement and Rheumatic Heart Disease

**DOI:** 10.3389/fped.2014.00148

**Published:** 2015-01-21

**Authors:** Marcia Holsbach Beltrame, Sandra Jeremias Catarino, Isabela Goeldner, Angelica Beate Winter Boldt, Iara José de Messias-Reason

**Affiliations:** ^1^Department of Clinical Pathology, Hospital de Clínicas, Universidade Federal do Paraná, Curitiba, Brazil; ^2^Department of Genetics, Universidade Federal do Paraná, Curitiba, Brazil

**Keywords:** lectin pathway, complement system, MBL, ficolins, gene polymorphisms

## Abstract

The innate immune system is the first line of host defense against infection and is comprised of humoral and cellular mechanisms that recognize potential pathogens within minutes or hours of entry. The effector components of innate immunity include epithelial barriers, phagocytes, and natural killer cells, as well as cytokines and the complement system. Complement plays an important role in the immediate response against microorganisms, including *Streptococcus* sp. The lectin pathway is one of three pathways by which the complement system can be activated. This pathway is initiated by the binding of mannose-binding lectin (MBL), collectin 11 (CL-K1), and ficolins (Ficolin-1, Ficolin-2, and Ficolin-3) to microbial surface oligosaccharides and acetylated residues, respectively. Upon binding to target molecules, MBL, CL-K1, and ficolins form complexes with MBL-associated serine proteases 1 and 2 (MASP-1 and MASP-2), which cleave C4 and C2 forming the C3 convertase (C4b2a). Subsequent activation of complement cascade leads to opsonization, phagocytosis, and lysis of target microorganisms through the formation of the membrane-attack complex. In addition, activation of complement may induce several inflammatory effects, such as expression of adhesion molecules, chemotaxis and activation of leukocytes, release of reactive oxygen species, and secretion of cytokines and chemokines. In this chapter, we review the general aspects of the structure, function, and genetic polymorphism of lectin-pathway components and discuss most recent understanding on the role of the lectin pathway in the predisposition and clinical progression of Rheumatic Fever.

## Complement System: An Overview

The high complexity of the human immune system provides not only effective defense against an impressive number of pathogens but also protection to undesirable response against self-components. The immune system is classically divided in two parts, the innate and the adaptative, which are wide-ranging and interconnected. The innate immune system provides an immediate and non-specific first line defense through humoral, cellular, and mechanical processes, playing a vital role in protection against pathogenic challenge (1). The complement system is an essential part of the innate immune system, with three overlapping roles: defense against infection, clearance of immune complexes and cell debris, and link between innate and adaptative immunity (2).

**Figure 1:**
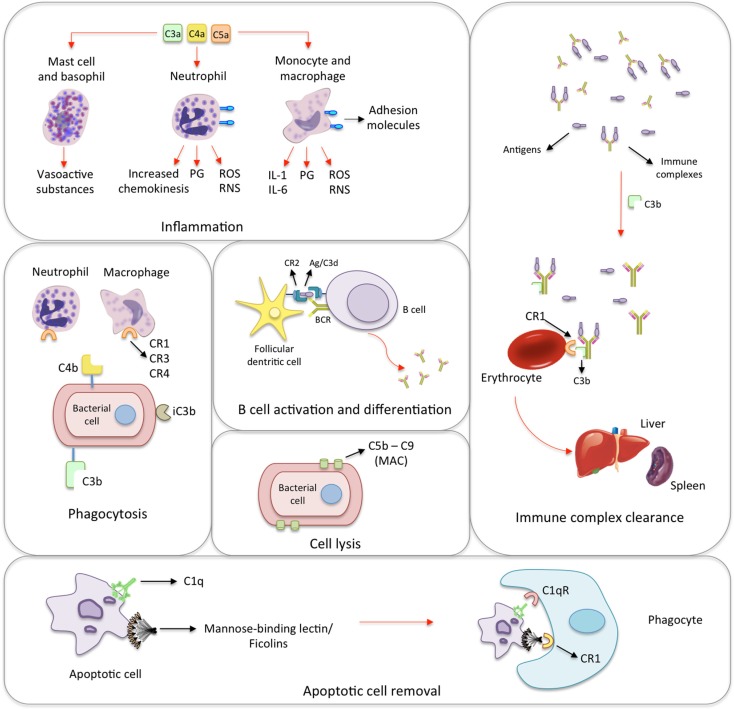
**Biological functions of the complement system**. **Inflammation**: the activation of the complement system generates many anaphylatoxins, among which C3a, C4a, and C5a. The binding of C3a, C4a, and C5a to receptors on mast cells and basophils leads to the release of histamine and other vasoactive mediators. In response to the activation by anaphylatoxins, neutrophils release prostaglandins (PG), reactive oxygen and nitrogen species (ROS and RNS, respectively) as well as increase the expression of adhesion molecules, and chemokinesis. Monocytes and macrophages show similar response and secrete interleukins 1 and 6 (IL-1 and IL-6). **Phagocytosis**: iC3b, C4b, and mainly C3b coat microorganisms and immune complexes, having opsonizing activity. Neutrophils and macrophages express complement receptors (CR1, CR2, and CR4), which bind C3b, C4b, and iC3b. This promotes the adherence of the microorganism to phagocytic host cells leading to phagocytosis. **B cell activation and differentiation**: the recognition of C3-tagged antigen plays an important role in B cell activation and differentiation. Cross-linking between complement receptor 2 (CR2) and B cell receptor (BCR) through C3d–antigen complexes lowers the threshold of B cell activation leading to migration, T cell/B cell interaction and antibody class-switch. **Cell lysis**: specific antibodies, MBL/ficolins, and spontaneous hydrolysis of C3 activate the complement on the surface of infectious microorganisms and lead to the formation of membrane-attack complexes (MAC), which cause their lysis. **Immune complex clearance**: immune complexes activate the complement system. The generated C3b binds to the complexes and to CR1 present on the surface of erythrocytes. During erythrocyte traffic through sinusoids in liver and spleen, resident phagocytes remove bound immune complexes leading to their clearance. **Apoptotic cell removal**: Mannose-binding lectin, ficolins and C1q bind debris of apoptotic cells, which are subsequently removed through binding to the C1qR and CR1 receptors on phagocytic cells.

The complement system consists of more than 35 plasma proteins and cell surface complement receptors and regulatory proteins. Most of the soluble proteins circulate in functionally inactive forms as proenzymes or zymogens (3). Upon proteolytic cleavage, inactive molecules become activated resulting in a proteolytic cascade that elicits a number of effector functions including phagocytosis, inflammation, cell lysis, and guidance of the adaptative immune response (Figure 1) (4). Since activation of complement leads to potentially destructive effects, several inhibitors tightly regulate this system in order to protect host tissues (5). Thus, an effective performance of the complement system depends on balancing regulatory and activation mechanisms, focused on destroying invading microorganisms and limiting damage of host cells and tissues. The imbalance on this fine equilibrium leads to harmful effects to the host, with potentially severe outcomes (2).

**Figure 2:**
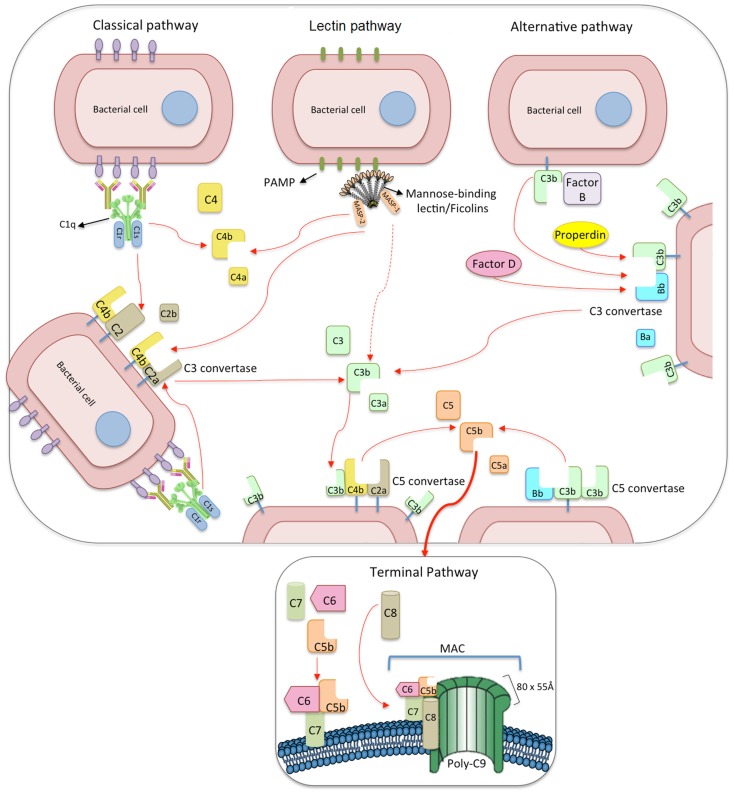
**The three pathways of complement activation: classical, lectin, and alternative pathways**. The classical pathway is initiated via binding of C1 complex (which consists of C1q, C1r, and C1s molecules) through its recognition molecule C1q to antibody complexes on the surface of pathogens. Subsequently, C1s cleaves C4, which binds covalently to the pathogen surface, and then cleaves C2, leading to the formation of C4b2a complex, the C3 convertase of the classical pathway. Activation of the lectin pathway occurs through the binding of the complex of mannose-binding lectin (MBL), CL-K1 or ficolins, and MBL-associated serine proteases 1 and 2 (MASP-1 and MASP-2, respectively) to various carbohydrates or acetylated residues on the surface of pathogens (PAMP, pathogen-associated molecular pattern). Like C1s, MASP-2 leads to the formation of the C3 convertase, C4b2a, but its activation is dependent on MASP-1. MASP-1 also cleaves C2 and C3. Activation of the alternative pathway depends on spontaneous low-grade hydrolysis of C3 in plasma leading to the formation of C3b. This C3b binds factor B (homologous to C2) to form a C3bB complex. The cleavage of factor B by factor D form the alternative pathway C3 convertase, C3bBb. Properdin stabilizes this complex. The C3 convertases cleave C3 to C3b, which binds covalently next to the site of complement activation (opsonization). This amplifies the cascade and mediates phagocytosis, as well as adaptative immune responses. The addition of further C3b molecules to the C3 convertase forms C5 convertases (C3bBbC3b for the alternative pathway or C4bC2aC3b for both classical and lectin pathways), initiating the assembly of the membrane-attack complex (MAC) by cleavage of C5 to C5a and C5b. Whereas C5a functions as a potent anaphylatoxin, C5b forms a complex with C6 and C7, which is inserted in the cell membrane. Thereafter, C8 and 10–18 C9 molecules (80 × 55 Å each) bind to this complex, resulting in a fully functional MAC (C5b-9). The three pathways converge to this common terminal pathway, culminating with cell lysis and death.

The activation of complement can take place on the surface of pathogens or damaged/infected cells by three distinct but converging cascade pathways: classical, alternative, and lectin. All three pathways are initiated by multiple stimuli independently from each other and subsequently the proteolytic cascades converge toward the activation of the major component C3, which results in the assembly of the membrane-attack complex (MAC) (Figure 2) (5). The activation of the classical pathway is initiated on immune complexes by the binding of C1q to Fc portion of IgM or IgG (6, 7). On the other hand, the activation of the alternative pathway occurs by spontaneous hydrolysis of C3 in plasma (6). Similarly to the alternative pathway, the lectin pathway may be activated in the absence of immune complexes. It is initiated by the binding of pattern-recognition plasma molecules such as mannose-binding lectin (MBL), collectin 11 (CL-K1), or ficolins to carbohydrates or acetylated residues present on microorganisms or to aberrant glycocalyx patterns on apoptotic, necrotic, or malignant cells (7). The lectin pathway also plays a role in the coagulation system through the binding of MBL–MBL-associated serine proteases (MASPs) or ficolin–MASPs complexes to fibrinogen or fibrin (8).

## Lectin Pathway of Complement Activation

The lectin pathway is initiated when pattern-recognition molecules (MBL, CL-K1, and ficolins) bind to the so-called pathogen-associated molecular patterns (PAMPs) (D-mannose, N-acetyl-D-glucosamine, or acetyl groups), on the surface of pathogens or to apoptotic or necrotic cells (9).

Circulating MBL, CL-K1, and ficolins form complexes with two dimers of MASPs, MASP-1 and MASP-2. After the binding of MBL/MASPs, CL-K1/MASPs, or ficolin/MASPs complexes to their targets, MASP-1 can auto-activate and trigger MASP-2 (10), leading to C4 and C2 cleavage. This allows the assembly of the C3 and C5 convertases, with subsequent activation of C3 and C5, respectively, and generation of C3a and C5a, two pro-inflammatory anaphylatoxins that increase the inflammatory response. The fragment C3b binds covalently to hydroxyl and amino groups on the surface of target molecules of all three pathways. In the absence of complement regulatory proteins, a powerful amplification in the number of surface-bound C3b molecules takes place through the alternative pathway. In this amplification loop, factor B binds to the attached C3b and is cleaved by factor D generating the alternative pathway C3 convertase C3bBb, which leads to accelerated C3b formation (11, 12). C3b tags antigens/pathogens for opsonization and antigen presentation or killing by phagocytes through the interaction with complement membrane receptors CR1, CR2, CR3, and CR4, and the immunoglobulin superfamily member CRIg (13). Finally, the complement cascade culminates with the formation of the multiprotein complex (C5b, C6, C7, C8, and C9_n_) known as terminal complement complex or MAC, which are inserted as pores of up to 11 nm into the cell membrane inducing loss of membrane integrity and ultimately cell death (14, 15) (Figure 2).

The following topics will cover the main components of the lectin pathway, their functions, polymorphisms, and relevance on the susceptibility to rheumatic fever (RF) and rheumatic heart disease (RHD).

### Mannose-binding lectin

Mannose-binding lectin is a central recognition molecule of the lectin pathway, synthesized in liver cells and secreted into bloodstream as high molecular weight multimeric complexes (16). It is a member of the collectin family of proteins, sharing collagen, and carbohydrate-recognition domains (CRD). MBL is known as a C-type lectin due to the ability to recognize sugar moieties in a Ca^2+^-dependent manner, and is also referred as “defense collagen” because of the important role in the innate immunity and pathogen clearance (17).

**Figure 3:**
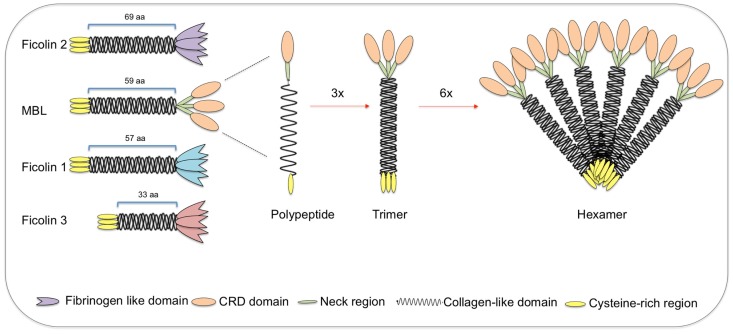
**Structural subunits of mannan-binding lectin (MBL) and ficolins**. Both MBL and ficolins contain a short N-terminal cysteine-rich region followed by a collagen-like sequence [length given in number of amino acids (aa)]. The C-terminal region is a carbohydrate-recognition binding domain for MBL (shown as oval forms) and a fibrinogen-like domain for ficolins (shown as tulip forms). The polypeptides interact through its collagen-like region forming triple helices (trimers), which further associate into higher oligomeric arrangements (tetramers to hexamers). Despite the very different structures, ficolin polypeptides and trimers interact in the same way as MBL, forming high oligomeric forms (tetramers). MBL-associated serine proteases interact with the collagen-like region, thereby activating the lectin pathway of complement.

Mannose-binding lectin is basically formed by a trimer of identical polypeptide chains, each containing a cysteine-rich N-terminal domain, a collagen-like region, an alpha-helical coiled-coil neck domain, and a C-terminal CRD (18). The three chains are associated by disulfide bonds and form the structural unit of MBL, which, in turn, polymerize into higher-order MBL oligomers (19) (Figure 3). Single MBL trimers are not fully functional, in contrast to dimers and higher-order oligomers, with tetramers predominating in the circulation (20). The recognition and binding of MBL to its ligands occurs through the CRD domain, and the oligomeric configuration confers multivalent and high avidity binding to targets (21, 22).

Mannose-binding lectin recognizes repetitive arrays of carbohydrate structures on pathogenic organisms such as viruses, bacteria, fungi, protozoans, and multicellular parasites as well as on apoptotic/tumoral cells (23–27). And despite its name, MBL does not bind selectively only mannose or its multimers, but rather recognizes sugars with 3- and 4-OH groups placed in equatorial plane of the sugar ring, which include glucose, L-fucose, *N*-acetylmannosamine (ManNAc), and *N*-acetylglucosamine (GlcNAc), but not galactose (28). MBL can further bind phospholipids (29), nucleic acids (30), and non-glycosylated proteins (31).

After binding to targets, MBL induces several biological effects such as complement activation by the lectin pathway, opsonophagocytosis, modulation of inflammation, and recognition of altered self-structures (32). In addition, MBL may modulate cytokine production at both mRNA and protein levels (33).

Mannose-binding lectin also plays a role in the clearance of apoptotic cells by recognizing damage-associated molecular patterns (DAMPs) (34). By binding to terminal sugars of cytoskeletal proteins in apoptotic cells, MBL mediates their recognition and phagocytosis by macrophages leading to their clearance. In fact, both C1q and MBL facilitate the binding of apoptotic cells to immature dendritic cells as well as to macrophages (27). Defects in the clearance of apoptotic cells have been implicated in the pathogenesis of some autoimmune conditions, although the precise role of MBL in this process remains unknown. Experiments using MBL-deficient mice showed impaired removal of apoptotic cells but no relation to autoimmune disease (35).

### MBL serum levels and *MBL2* gene polymorphisms

Mannose-binding lectin is encoded by the *MBL2* gene, located on the long arm of chromosome 10 (10q11.2–q21) (36). It is considered an acute-phase reactant (37), whose levels can increase up to threefold during the acute-phase response, mainly due to up-regulation by acute-phase mediators (38). MBL serum levels range from a few nanogram per milliliter to more than 10 μg/ml, with a mean value of around 0.8 μg/ml (39). However, MBL levels are largely dependent on *MBL2* genetic polymorphisms, which are responsible for inter-individual variations of up to 10-fold on circulating MBL levels (40, 41). Besides genetic variation, MBL levels may also vary significantly during lifetime (42–44).

Mannose-binding lectin deficiency is fairly common, affecting approximately 8–10% of individuals and usually defined as ≤100 ng/ml in the circulation (45, 46). MBL deficiency is more harmful when there are additional co-existing immune defects (47), since the majority of MBL-deficient individuals are essentially healthy (48). MBL deficiency has been associated with upper respiratory tract infections in young children and with the susceptibility to severe infections in patients receiving chemotherapy (45). However, it may be beneficial in infections due to intracellular pathogens, such as *Mycobacterium* spp. and *Leishmania chagasi*, which use C3 opsonization and C3 receptors to invade host cells (49–52).

*MBL2* is a highly polymorphic gene, exhibiting variants responsible for large variations in both MBL levels and functional activity (53–57). These variants include SNPs located in the first exon of *MBL2* gene, being at least one synonymous SNP (on codon 44 for asparagine) and eight non-synonymous variants (including B, C, and D, which are detailed in the next paragraph). At least other three SNPs located in the promoter region of the *MBL2* gene also have influence on MBL levels, called MBL2*H and *L* alleles (due to a polymorphism located at −550 bp), *X* and *Y* alleles (a SNP at −221 bp) and *P* and *Q* alleles (a non-coding SNP at +4 bp), all positions counted from the transcription start site (58, 59).

**Figure 4:**
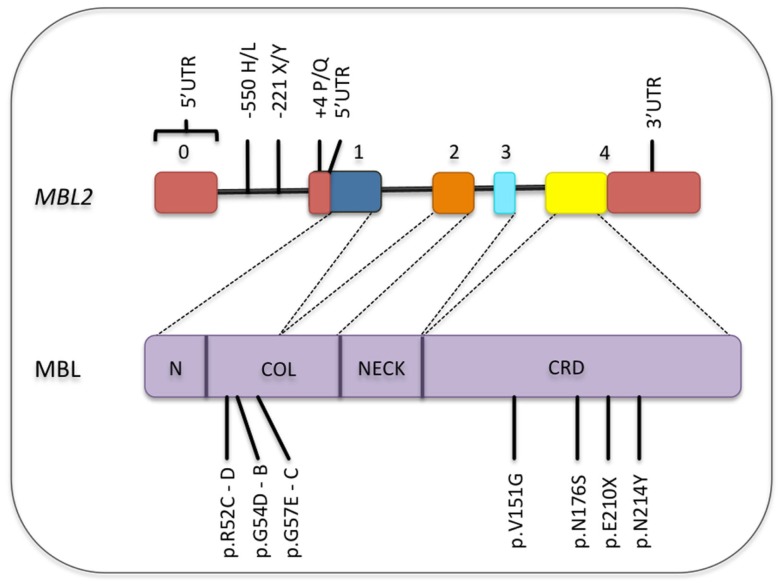
**Common polymorphisms in the *MBL2* gene and its corresponding locations in the MBL protein**. Only the functional polymorphisms in the promoter and non-synonymous mutations are shown [SNP database and Boldt et al. (161)]. Exons are numbered. Exons, introns, and protein domains are not in scale. N, N-terminal region; COL, collagen-like region; CRD, carbohydrate-recognition domain.

In 1991, Sumiya et al. sequenced the complete *MBL2* gene in three British children with recurrent bacterial infections and low MBL levels. All of them had the *B* allele (an exon 1 point mutation at codon 54, changing *GGC* to *GAC* and causing an amino acid change of glycine to aspartic acid – *p.Gly54Asp*) (60). Others subsequently found two other deficiency-causing common substitutions, allele *D* in codon 52 (*CGT* to *TGT*), changing arginine to cysteine (*p.Arg52Cys*) and allele *C*, in codon 57 (*GGA* to *GAA*), substituting glycine for glutamic acid (*p.Gly57Glu*) (61) (Figure 4). Exon 1 mutations dramatically reduce protein assembly and stability, increasing the amount of poorly oligomerized MBL with reduced capacity of complement activation and ligand binding in homozygous (e.g., *B/B*) or compound heterozygous (e.g., *B/C*) carriers (18). The wild allele at these loci is called *A*, whereas *D*, *B*, and *C* alleles have been collectively called *0*. While *0*/*0* individuals have near undetectable levels of high-order MBL oligomers, *A/0* individuals may present reduced plasma protein levels (61, 62). In addition, a promoter variant 221 bp before the start of transcription site, with *X* and *Y* alleles (*g.602G* > *C*), markedly decreases levels of otherwise fully functional MBL proteins.

### Ficolins

Similarly to MBL, ficolins are pattern-recognition receptors that are able to associate with MASPs and activate the complement system through the lectin pathway, having an essential role in the immune defense against clinically important pathogens. Besides activating complement, they limit infection by stimulating the secretion of interferon gamma (IFN-γ), IL-17, IL-6, tumor necrosis factor alpha (TNF-α), and nitric oxide (NO) by macrophages (63).

Ficolins form oligomers of four structural subunits joined together via disulfide bridges at the N-terminal regions, similarly to MBL, but higher or smaller oligomers seem to be less common for ficolins (21). They should not be referred as lectins (meaning that carbohydrates are the preferred ligands for lectins), since they target acetylated compounds relatively independently of the structure of the acetylated molecule (64).

Three human ficolins have been described, each encoded by its own gene. Ficolin-1 can be found attached to cell membrane or soluble in plasma, with a concentration of 0.05–1.0 μg/ml. It is also found in the secretory granules of monocytes, gelatinase granules of neutrophils, and type II alveolar epithelial cells (65–68). Ficolin-1 recognizes common acetylated compounds, including GlcNAc and GalNAc (63, 67, 69, 70) binding to several microorganisms including Gram-positive (*Staphylococcus aureus*) and Gram-negative bacteria (*Salmonella typhimurium* LT2) (67, 69). It is the only human ficolin able to bind to sialic acid, found on capsular polysaccharides of some pathogens such as *Streptococcus agalactiae* as well as on the surface of immune cells. Thus, Ficolin-1 is supposed to play a role in the modulation of immune cell interaction (71) and in blood coagulation and/or fibrinolysis (72). Importantly, Ficolin-1 and pentraxin-3 heterocomplexes act as non-inflammatory signals, promoting clearance of altered self-cells and modulating IL-8 production (73).

*FCN1* gene is located on chromosome 9q34 (74) and contains nine exons. Among the several SNPs described for the *FCN1* gene, at least eight are associated with Ficolin-1 levels, four of them located in the promoter and in the first exon (74). These polymorphisms are partly responsible for the wide range (up to 15-fold) of inter-individual variability in Ficolin-1 plasma concentrations (66). *FCN1* polymorphisms were associated with increased fatal outcome in patients with systemic inflammation (75) and to the susceptibility to rheumatoid arthritis (76). Low Ficolin-1 levels have been associated with a 12-fold increased risk of fatal necrotizing enterocolitis, and with need for mechanical ventilation (77), as well as with occurrence of severe infections in cancer patients undergoing chemotherapy (78).

Ficolin-2 is a plasma protein, which is mainly produced in the liver (79), but low mRNA levels were also found in the bone marrow, intestine, tonsils, and fetal lung (80). In Europeans, the median plasma concentration is around 5 μg/ml, with inter-individual variations ranging from 1 to 12 μg/ml (81). Ficolin-2 is able to bind *N*-acetylated molecules, such as Acetyl-d-glucosamine (GlcNAc), *N-*acetylgalactosamine, and *N*-acetyllactosamine (71, 82), as well as artificially acetylated compounds (83). It binds also *N*-acetylneuraminic acid present on encapsulated opportunistic pathogens such as Group B streptococci (*Streptococcus agalactiae*) (84), bacterial peptidoglycan (PGN), fungal 1,3-beta-D-glucan (85), and envelope glycoproteins of hepatitis C virus (86). In addition, Ficolin-2 was shown to bind to *Mycobacterium bovis* (87) and flagellated protozoa including *Giardia intestinalis* (88) and *Trypanosoma cruzi* (89) and to interact with C-reactive protein, stabilizing its binding to bacteria and thereby activating complement (63, 90).

*FCN2* gene is located on chromosome 9q34.3 (79), with three SNPs in the promoter region and one in exon 8 being associated with variation in Ficolin-2 plasma levels: −*986G* > *A*, −*602G* > *A*, and −*4A* > *G* and *p.Ala258Ser*, while two other SNPs, at positions −557 and −64, appear not to influence gene expression (91, 92). Ficolin-2 insufficiency (<1,200 ng/ml) (93) has been associated with bronchiectasis and respiratory infection especially when co-existing with atopic disorders (94–96), but did not affect susceptibility to invasive pneumococcal disease (97). Low Ficolin-2 levels were also related with prematurity, low birth weight, and perinatal infections in neonates from Poland (98) and with the susceptibility to chronic Chagas disease (99). And although not associated with the development of malaria, children with severe malaria presented higher levels of Ficolin-2 than children with the mild form of the disease (100). On the other hand, *FCN2* polymorphisms associated with normal Ficolin-2 levels had a protective effect against the susceptibility to leprosy (101).

Ficolin-3 is the most abundant recognition molecule of the lectin-pathway with a mean plasma concentration around 18.4 μg/ml (102), varying approximately 10-fold among individuals (3–54 μg/ml) (103). Ficolin-3 was found highly expressed in the liver and lung tissues, indicating its significance in both activation of the lectin pathway and pulmonary host defense (80, 104). For that reason, Ficolin-3 is considered to play an important role in both systemic and local innate immune responses (80, 105, 106). Ficolin-3 was shown to recognize acetyl groups present in a wide range of microorganisms, including *Salmonella typhimurium*, *Salmonella minnesota*, *Escherichia coli*, and *Aerococcus viridans* (107, 108). It was also shown to share binding sites with Ficolin-2 and MBL on the surface of *Giardia intestinalis* (88). Furthermore, Ficolin-3 may mediate the clearance of late apoptotic cells and may have a beneficial role against autoimmunity (109, 110).

*FCN3* gene is located on chromosome 1p36.11 and is highly conserved in humans. Five amino acid exchanges were described, all with allele frequencies below 5%: *p.Leu12Val*, *p.Leu117fs*, *p.Thr125Ala*, *p.Glu166Asp*, and *p.Val287Ala* (80). This high conservation indicates that Ficolin-3 might exert crucial function in the immune response. Indeed, Ficolin-3 insufficiency is extremely rare (111) and was found associated with necrotizing enterocolitis in premature neonates (112).

### MBL-associated serine proteases

MBL-associated serine proteases act as activators of the lectin pathway upon binding of MBL, ficolins, and CL-K1 to carbohydrates or acetyl groups on the surface of pathogens or altered self-tissues (4). So far, five proteins have been identified, including three MASP enzymes (MASP-1, MASP-2, and MASP-3) and two truncated proteins, MAp19 and MAp44, which lacks the serine protease domain and consequently, functional activity (113, 114). All MASPs are capable to associate with MBL, ficolins, and CL-K1 in the presence of Ca2+, forming a proteolytic complex (39).

Both MASP-1 and MASP-2 play a crucial role in the activation of lectin pathway. Recent studies showed that MASP-1 can auto-activate and lead to MASP-2 activation (115, 116). MASP-2 can also auto-activate, but under physiological conditions, MASP-1 is the essential MASP-2 activator (39). MASP-2 is a protease that cleaves very efficiently C4 and C2, generating C3 convertase (113, 117). On the other hand, MASP-3 seems to attenuate the lectin-pathway activity due to competition for MASP binding sites on the recognition molecules (118). In addition, MASP-3 occurs predominantly complexed with Ficolin-3 and is thought to have an inhibitory effect on complement activation mediated by Ficolin-3 (119). MASP-3 also participate on developmental processes (120). The roles of MAp19 and MAp44 are still not well understood, but MAp44 was shown to negatively regulate the lectin pathway by competing for the same binding sites of MASP-2 and MASP-1 (121, 122).

MASP-1 was the first MASP to be reported (123). While MASP-1 and MASP-2 are produced mainly in the liver and present in plasma at 11 and 0.4 μg/ml, respectively (124, 125), MASP-3 is produced in several other tissues besides the liver (118). All three MASPs are structurally similar to each other and to both C1r and C1s. MASP-1, MASP-3, and MAp44 are codified by *MASP1* gene on chromosome 3q27–q28 and MASP-2 and MAp19 are encoded by *MASP2* gene located on chromosome 1p36.23–31 (119, 121, 126, 127). Some polymorphisms on *MASP1* and *MASP2* genes lead to changes in serum levels and functions of MASPs, thereby influencing complement activation by the lectin pathway (128, 129). Ammitzbøll et al. found 10 SNPs in the *MASP1* gene that were associated with serum levels of MASP-1, MASP-3, and MAp44 (130).

*MASP1* polymorphisms were associated with 3MC syndrome (131–133) and to *Pseudomonas aeruginosa* colonization in cystic fibrosis patients (134). Moreover, MASP levels (MASP-1, MASP-2, and MASP-3) were shown as a predictor for infection and prolonged dependency of intensive care in critically ill children (135). On the other hand, *MASP2* polymorphisms were associated with the susceptibility to leprosy (136), human T lymphotropic virus infection (137), malaria (138), Chagas disease (139), bacterial infections (140), and hepatitis C (141). MASP-2 levels have been related also with a number of diseases, including schizophrenia (142), septic shock (143), acute lymphoblastic leukemia, non-Hodgkin lymphoma, central nervous system tumors (144), colorectal cancer (145, 146), among others. Taken together, these studies have provided evidence for an emerging and important biological role of MASPs in human diseases.

## Lectin Pathway in RF and RHD

The lectin pathway appears to be involved in the pathophysiology of different cardiac conditions (147), sometimes with opposite roles or even ambiguous functions. Although low MBL producing genotypes were associated with coronary artery disease in American Indians (148), elevated MBL levels were associated with an increased risk of future coronary artery disease in men, but not in women, in the United Kingdom (149). In addition, MBL was shown to have an ambiguous role in the development of coronary artery lesions in Kawasaki disease, being protective in infants but potentially harmful in older patients (150). A protective role for high MBL levels were reported in myocardial infarction, particularly in diabetes (151), and *MBL2* variants related to functional MBL deficiency were shown to increase by twofold the risk of myocardial infarction in healthy individuals (152). Also, MBL levels measured 1 month after acute myocardial infarction were inversely associated with the incidence of reinfarction, suggesting that low MBL levels could predispose to ischemic events (153). However, experimental studies showed involvement of MBL in ischemia/reperfusion injury, probably due to its ability to recognize altered self-structures, thereby mediating complement activation (154, 155). Absence of MBL/MASP pathway activation has protected against tissue damage and preserve cardiac function in these disease models (154). In addition, inhibition of MBL by monoclonal antibodies decreased significantly the infarcted size and tissue injury by limiting neutrophil infiltration and gene expression of pro-inflammatory mediators (155). In fact, increasing evidence is indicating a pro-inflammatory role for MBL in chronic diseases and situations where there is undesirable complement activation and tissue injury (49–51).

Ficolins and MASPs were found to play a role as well in myocardial infarction. Ficolin-2 and Ficolin-3 levels were associated with left ventricular dilatation after myocardial infarction (156) and with advanced heart failure and outcome, respectively (157). Recent study involving all MASPs showed that MASP-1 levels were highest in subacute myocardial infarction and lowest in acute stroke patients, while MASP-2 levels were low in both conditions and MASP-3 and MAp44 levels did not differ between the groups. On the other hand, MASPs and MAp44 levels were associated with cardiovascular risk factors including dyslipidemia, obesity, and hypertension in patients with stable coronary artery disease (158). Moreover, MASP-2 levels were found significantly reduced in myocardial infarction, probably due to the activation of the lectin pathway during acute myocardial ischemia (159).

Despite the important role of the lectin pathway in complement activation and host defense against infection and autoimmunity, studies on the significance of components of this pathway in RF and RHD are still scarce. Different groups investigated the role of MBL and ficolins in the innate immunity against streptococcal infection. MBL binds strongly to N-acetyl-β D-glucosamine on the streptococcal cell wall and thereby promotes complement deposition and opsonization *in vitro* (160). Thus, MBL could play an essential role in the first steps of immune defense against streptococci infection, leading to complement activation, and pathogen phagocytosis (161). In addition, Ficolin-2 binds molecular patterns such as lipoteichoic acid on Gram-positive bacteria cell wall, including *Streptococcus pyogenes* (162), also mediating bacteria opsonization and elimination (79). Moreover, a critical role for ficolins in the protection against *Streptococcus pneumoniae* infection was shown in experimental models using ficolin deficient mice, supporting the contribution of these pattern-recognition molecules in the immune defense during streptococci infection (163).

Inherited MBL insufficiency, which leads to impaired innate immune function and enhanced susceptibility to infection, is essentially caused by three structural variants in exon 1 of *MBL2* gene. These polymorphisms include the previously mentioned variations in the collagenous tail, with the alleles being designated, respectively, *D*, *B*, and *C* (collectively termed “*0*”). In addition, another promoter variant identified as X/Y (*g.602G* > *C*) is known to significantly reduce circulating levels of functional MBL. In an initial study in 2001, there was no association between the *A*, *B*, *C*, and *D* alleles and RHD in Chinese patients (164). However, the authors suggested a putative role for MBL deficiency in the progression of RHD, by considering the age of onset of heart disease. The mean age of onset of cardiac symptoms of patients with the deficient *B* allele was significantly lower compared with patients with *MBL2* genotype *A/A* (30 ± 14 vs. 37 ± 11 years, *p* < 0.05). These results supported the hypothesis that MBL deficiency caused by the *B* allele could facilitate the development of RHD in younger people and accelerate the progression of RHD. Another study reported lower MBL serum levels in RHD patients from Yemen, compared with blood donors (165).

On the other hand, Schafranski et al. showed that MBL levels were significantly high in patients with RHD from South Brazil and that MBL deficiency was more prevalent among controls. The authors suggested that high MBL levels could be a cause of undesirable complement activation in RHD patients, contributing to the pathogenesis of rheumatic cardiomyopathy (166). Subsequent analysis on *MBL2* polymorphisms in the same cohort of patients showed that *MBL2* genotypes associated with high MBL levels were also significantly associated with RHD, if compared with controls, suggesting a role for high-producing *MBL2* genotypes in the susceptibility to RHD. Moreover, *MBL2 A/A* genotypes were significantly associated with higher MBL levels in RHD patients and not in controls. Homozygosity for the MBL2*A allele as well as for haplotypes associated with high MBL levels were associated with increased risk of RHD. Conversely, the frequency of *MBL2* variant alleles as well as *0/0* genotypes, which are associated with MBL deficiency, were lower in patients (167). These findings indicated that both *MBL2* alleles and protein levels were associated with susceptibility to RHD. Since MBL is a key molecule in the innate host defense against bacterial infection, the authors postulated that MBL could be considered as a double-edged sword molecule in the physiopathology of RF and RHD, on one hand conferring protection against initial infection by rheumatogenic streptococci, but on the other hand eliciting inflammation and complement tissue damage in the chronic stage of the disease (167). Later on, these findings were confirmed by showing that high MBL levels (>2,800 ng/ml) increased the risk of RHD and that genotypes associated with high MBL production (*YA/YA* and *YA/XA*) were associated with both acute and chronic rheumatic carditis (168). The results led to the conclusion that high MBL levels and its associated genotypes could be involved in development of cardiac manifestation in RF, and the association was due to genetic influence of *MBL2* polymorphisms rather than to an acute-phase reaction. The authors suggested that genotypes associated with high MBL production may represent a risk factor for development of acute and chronic rheumatic carditis in RF, and MBL could be involved in the ongoing inflammatory process and progression to the chronic cardiac form (168). Under stressful conditions such as inflammatory processes mediated by oxidative stress, self-cell surfaces may become glycosylated and thereby acting as targets for MBL/ficolin binding. This in turn, would lead to pathological complement activation and inflammation (169). In fact, myxomatous tissue rich in hyaluronic acid was observed in heart valves of RHD patients. Since *N*-acetyl-d-glucosamine is a ligand for MBL and a major constituent of hyaluronic acid in myxomatous tissue, activation of complement in RF may occur partially due to the binding of MBL to neo exposed ligands in the heart valves (170). Only the long-term follow-up of patients with acute RF without valve sequel would answer this question and confirm if in fact, high MBL levels and associated genotypes are risk factors to carditis in RF. Considering that no effective treatment is currently available for rheumatic carditis, studies exploring anti-MBL blocking in experimental models of RF and RHD could bring new insights in to this question and might unveil alternatives for future treatment of this disease.

Another study on patients with chronic severe aortic regurgitation (AR) of rheumatic etiology by Ramasawmy et al. showed that MBL2*D allele frequency was higher among AR patients than in controls from São Paulo, Brazil. The *B* and *C* alleles had similar frequencies in both groups, whereas the frequency of the *0/0* genotypes was significantly different between patients and controls, but similar in both groups for *A/0* heterozygotes. Thus homozygous or compound heterozygous patients for defective *MBL2* alleles (genotype *0/0*) presented higher risk of developing chronic AR. In this case, the authors postulated that continuous exposure to *Streptococcus group A* antigens in MBL-deficient individuals would lead to subsequent abnormal immune response against heart proteins, leading to rheumatic AR (171). These results were different than previous reports (167, 168), in which *MBL2* wild-type *A* allele increased the risk to RHD in patients with mitral valve lesion of rheumatic etiology, rather than aortic vulvar lesion, as reported for AR patients. It is worth mentioning that the ancestry of patients differed significantly between the studies, whereas the AR patients from São Paulo were of mixed Brazilian ethnicity, those from the South were mainly Euro-Brazilians.

Some polymorphisms in *MASP2* gene were shown to impair the activation of the lectin pathway. A mutation in exon 3, *g.5620A* > *G*, leads to an amino acid substitution from aspartate to glycine (*Asp120Gly* or *p.D120G*) and disturbs calcium ion binding site in the first CUB1 domain of the mature protein. This defect causes loss of MASP-2 binding ability to MBL and ficolins, abolishing complement activation and decreasing MASP-2 circulating levels. The mutation *p.D120G* occurs in 0.15–0.3% of European individuals and has been reported to be the main cause for low MASP-2 levels in this population, with homozygosity resulting in MASP-2 deficiency (129, 172, 173).

In two independent studies, partial or total MASP-2 deficiencies resulting from *p.D120G* mutation were not associated with RF/RHD and severe chronic AR in Brazilian patients, respectively. No homozygotes but only heterozygotes for the *G* allele were found with no significant differences between patients and controls, indicating that the *p.D120G* variation in the *MASP2* gene does not have a relevant role in the pathogenesis of RF (171, 174). However, one must consider that the *G* allele is rare and only studies with a large number of subjects would have the statistical power to indicate a possible role for *p.D120G* in the development of RF or RHD (171, 172, 174–177). Recently, a number of different *MASP2* polymorphisms encompassing the promoter region to exon 12 and MASP-2 levels were investigated in RF and RHD patients. The authors found that low MASP-2 producing variants were associated with protection against the development of RHD and haplotypes sharing intron 9 – exon 12 polymorphisms increased the susceptibility to RHD, when compared to RF patients without cardiac disease. In addition, MASP-2 levels were lower in patients an associated with *MASP2* haplotypes. The results suggest a role for *MASP2* gene polymorphisms and protein levels in RHD (178).

**Figure 5:**
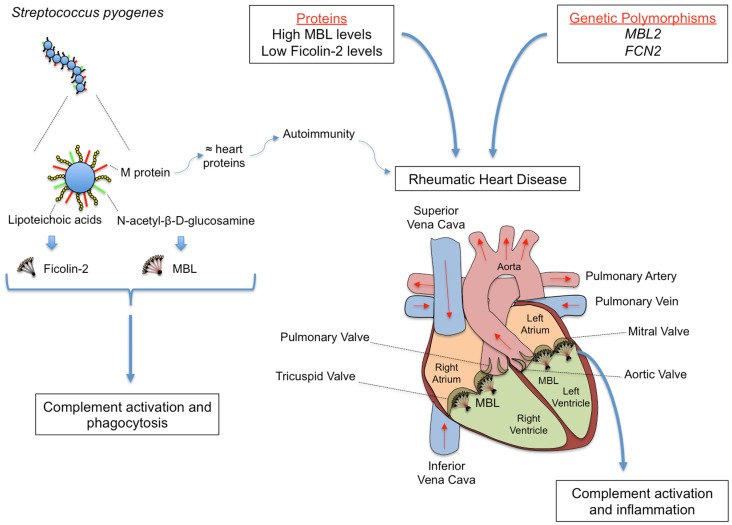
**Rheumatic fever (RF) and its most severe sequel chronic rheumatic heart disease (RHD) are chronic inflammations that follow oropharynx infection by *Streptococcus pyogenes*, whose cell wall presents several PAMPs, including M protein, lipoteichoic acid, and *N*-acetil-β-d-glucosamine**. M protein shares structural homology with heart proteins such as myosin and tropomyosin, leading to the formation of cross-reactive auto-antibodies. MBL and Ficolin-2 bind *N*-acetyl-β-d-glucosamine and lipoteichoic acid, respectively, inducing complement activation and phagocytosis. Although conferring protection against the initial infection, MBL may deposit on the altered valves, eliciting inflammation, and complement tissue damage in the chronic stage of the disease. The functional importance of the proteins may vary during infection and disease establishment, with *MBL2* and *FCN2* polymorphisms leading to high MBL and low Ficolin-2 levels, respectively, being associated with increased susceptibility to RHD.

The recognition molecules of the lectin pathway, including Ficolin-2, associate with MASPs in serum, forming complexes able to activate complement (179). Ficolin-2 presents a wide specificity for microorganisms, thereby having an important role in the first line of innate immune defense. Although clinical studies focusing on Ficolin-2 are still in their initial stages, there is evidence that Ficolin-2 deficiency might increase the risk of respiratory infections (180). So far, the only study on Ficolin-2 in RF/RHD investigated polymorphisms in the promoter region of the *FCN2* gene (at positions -986, -602, and -4) in patients and controls from South Brazil. The haplotype-986/-602/-4 *G/G/A*, associated with low Ficolin-2 levels, was significantly associated with RHD when compared to controls, suggesting that this haplotype may play a role in the progression of RF to its chronic form. On the other hand, the frequency of the *FCN2* haplotype-986/-602/-4 *A/G/A* was higher in the controls than in the patients (RF plus RHD), showing a protective effect against RF and RHD. This data led the authors to suggest that *FCN2* promoter haplotypes are associated with the susceptibility to RF and its chronic form RHD, with -986/-602/-4 *G/G/A* haplotype representing a novel risk factor for the susceptibility and clinical progression of the disease (181). The putative role of MBL and Ficolin-2 in RF and RHD is summarized in Figure 5.

In conclusion, the studies accomplished so far on the proteins and genes of the lectin pathway of complement pointed to an important role for the lectin pathway in the susceptibility to RF and clinical progression to RHD. However, future studies are required in order to clarify the role of the recognition molecules and serine proteases of the lectin pathway in RF and RHD.

## Conflict of Interest Statement

The authors declare that the research was conducted in the absence of any commercial or financial relationships that could be construed as a potential conflict of interest.
